# Effects of Olive and Pomegranate By-Products on Human Microbiota: A Study Using the SHIME^®^ In Vitro Simulator

**DOI:** 10.3390/molecules24203791

**Published:** 2019-10-21

**Authors:** Camilla Giuliani, Massimo Marzorati, Matteo Daghio, Andrea Franzetti, Marzia Innocenti, Tom Van de Wiele, Nadia Mulinacci

**Affiliations:** 1 Department of NEUROFARBA and Multidisciplinary Centre of Research on Food Sciences (M.C.R.F.S.-Ce.R.A.), University of Florence, Via Ugo Schiff 6, 50019 Sesto F.no Firenze, Italy; camilla.giuliani@unifi.it (C.G.); marzia.innocenti@unifi.it (M.I.); 2 Center for Microbial Ecology and Technology (CMET), Faculty of Bioscience Engineering, Ghent University, Coupure Links 653, 9000 Ghent, Belgium; massimo.marzorati@ugent.be (M.M.); tom.vandewiele@ugent.be (T.V.d.W.); 3 Department of Agriculture, Food, Environment and Forestry, University of Florence, Piazzale delle Cascine 18, 50144 Firenze, Italy; matteo.daghio@unimib.it; 4 Department of Earth and Environmental Sciences, University of Milano–Bicocca, Piazza della Scienza 1, 20126 Milano, Italy

**Keywords:** food by-products, human gut, microbial community, tyrosol, ellagitannins

## Abstract

Two by-products containing phenols and polysaccharides, a “pâté” (OP) from the extra virgin olive oil milling process and a decoction of pomegranate mesocarp (PM), were investigated for their effects on human microbiota using the SHIME^®^ system. The ability of these products to modulate the microbial community was studied simulating a daily intake for nine days. Microbial functionality, investigated in terms of short chain fatty acids (SCFA) and NH_4_^+^, was stable during the treatment. A significant increase in *Lactobacillaceae* and *Bifidobacteriaceae* at nine days was induced by OP mainly in the proximal tract. Polyphenol metabolism indicated the formation of tyrosol from OP mainly in the distal tract, while urolithins C and A were produced from PM, identifying the human donor as a metabotype A. The results confirm the SHIME^®^ system as a suitable in vitro tool to preliminarily investigate interactions between complex botanicals and human microbiota before undertaking more challenging human studies.

## 1. Introduction

In 2011, the Food and Agriculture Organization (FAO) of the United Nations reported that “one-third of the edible parts of food produced for human consumption gets lost or wasted globally, which is about 1.3 billion tons per year”. Post-harvesting and processing are responsible for more than 40% of losses, mainly in industrial countries [1]. In recent years, interest in food waste valorization and reuse has grown dramatically and more and more often these materials are more correctly defined as by-products. The goal of researchers and companies is to define new sustainable processes to efficiently recover bioactive compounds from normally discarded food by-products, and among the various matrices, those deriving from the processing of pomegranate and olive fruits are recognized as particularly interesting.

Different strategies have been proposed to manage wastewaters and pomaces derived from milling processes to reduce their negative effects, and also to recover the bioactive phenols [2,3,4,5]. It has been demonstrated that less than 1% of total phenolic compounds in olives is recovered in virgin oil during the milling process [6]. An interesting semisolid pomace from a two-phase olive mill is the by-product named pâté. This dry product showed antiaging activity in human cultured cells and can provide a high daily intake of phenolic constituents: approximately 1 g can contain the same amount of phenols present in 200 mL of a good quality extra virgin olive oil [7].

Analogously to olive oil by-products, the discarded parts of pomegranate fruit have also been the object of increased interest, particularly to recover the ellagitannins known as the typical constituents abundant in the external parts of the fruit discarded during juice production [8]. Several extractions have been proposed to maximize the recovery of ellagitannins [9] and polysaccharides [10] from the peel of the pomegranate, a by-product that can include up to 50% of the entire weight of fresh fruit [11].

It has been estimated that, after introduction into the organism through oral administration, only 5–10% of low molecular weight polyphenols are absorbed in the small intestine, mostly derived from deconjugation and deglycosylation reactions [12]. High molecular weight polyphenols, such as the ellagitannins, are not absorbed in the small intestine but reach the colon where they are metabolized [13]. The metabolism of polyphenols is strictly related to individual gut microbial composition. This variability leads to different enzymatic and metabolic pathways, which allow definition of a potential classification in enterotypes or nutritional phenotypes [14]. The advanced in vitro gastrosimulator system, SHIME, has already been applied to develop numerous studies focused on investigating the impact of different phenols on human microbiota [15,16]. The potential positive impact of phenolic compounds is strongly related to interindividual variability of microbial composition [17]. Polyphenols can influence bacterial growth and metabolism, depending on their structure, dose, and type of microorganisms considered [18], and can exert antimicrobial activity in a dose-dependent manner [19]. After exposure to polyphenols, microbial synthesis of defensive proteins increases, but at the same time their metabolic activity decreases, reducing the formation of biosynthetic proteins, amino acids, phospholipids, and short chain fatty acids (SCFAs) [13]. Furthermore, polyphenols exhibited antimicrobial activity against viruses, pathogenic bacteria, and fungi [20], but the mechanisms that lead to changes in the gut microbial community are strictly related to the chemical transformations that polyphenols undergo at the gut level. It has been widely demonstrated that there is a mutual interaction between the structural transformation of polyphenols performed by some bacteria and the different activities that such molecules should exert on the microbial community, since in some cases, the phenolic metabolites show higher antimicrobial activity or bioavailability with respect to their originators [21,22]. However, the in vitro systems currently applied in these studies reveal some limitations, since other aspects should be considered, such as the genetic and metabolic state of the host and the interaction of polyphenols with other dietary components [21,23]. Further efforts could be helpful to better clarify the mechanisms involved in the relationship between polyphenols and intestinal microbiota.

Gut microbiota has been recognized as a key factor in the health effects of pomegranate ellagitannins [24]; ellagic acid is released in the intestinal lumen by hydrolysis reactions of ellagitannins, and it is metabolized by bacteria in the large intestine and transformed into urolithins [25,26]. Urolithins have been found in fecal samples after oral administration of pomegranate juice [27], identifying three “enterotypes”, and also according to the urolithin profile in the urine [16,28].

Differently from pomegranate, the interaction of the main polyphenols from olive oil is still scarcely known, and there is little data regarding colonic transformation of these molecules [29,30,31,32].

The present study focuses on the effects on intestinal microbiota of two samples derived from well-known agricultural by-products using the advanced bowel simulator SHIME^®^. The final aim is to propose these by-products as potential sources of food ingredients with beneficial effects on human health. A further aim of this study is to sustain the suitability of a gastro-intestinal simulator such as SHIME^®^ for a preliminary evaluation of the safety of these new products. The obtained results in terms of toxicity, biotransformation, and mutual relationship between polyphenols and gut microbiota can be very useful to provide new insights before performing in vivo trials, which clearly have many limitations such as ethical and economic issues and require long times. The chosen samples obtained by simple and green procedures were an olive pâté (OP) recovered from extra virgin olive oil production and a decoction from pomegranate mesocarp (PM) of the Wonderful variety. The main objective of the study was to evaluate the effect of repeated feeding with PM or OP on human microbiota. The microbial functionality was investigated, determining SCFAs and NH_4_^+^ levels and microbiota composition by Illumina on bacterial DNA. Furthermore, polyphenols’ metabolic fate was studied by HPLC/DAD/MS analysis.

## 2. Results and Discussion

### 2.1. List of Abbreviations

Throughout the test, some abbreviations are used as references to define thedifferent samples. PCC is proximal colon control; DCC is distal colon control; PPCP is proximal colon olive pâté; DCOP is distal colon olive pâté day; PCPM is proximal colon pomegranate mesocarp; DCPM is distal colon pomegranate mesocarp. Day × referes to sampling day (0, 5, 9, 13).

### 2.2. Experiment Design and OP and PM Composition

The experiments were performed to investigate the mutual effects between the microbial community and the two samples that can be proposed as new functional ingredients in food formulation. Since human studies are difficult to manage mainly due to recruiting and compliance, ethical committee authorization, and real costs, the availability of a dynamic gastrointestinal simulator can be a very useful tool for preliminary screenings. The final aim of this study is to demonstrate that a possible use of OP and PM as new food ingredients is without risks (e.g., antimicrobial effects) for human microbiota.

The pomegranate extract is a combination of bioactive compounds containing ellagitannins and polysaccharides with in vitro prebiotic activity [10], a combination tested for the first time on the SHIME^®^ system. The olive extract selected in this work has already shown the ability to modify the microbiota when used as an additive in animal feed [33], but it has never been evaluated with human gut microbiota.

**Figure 1:**
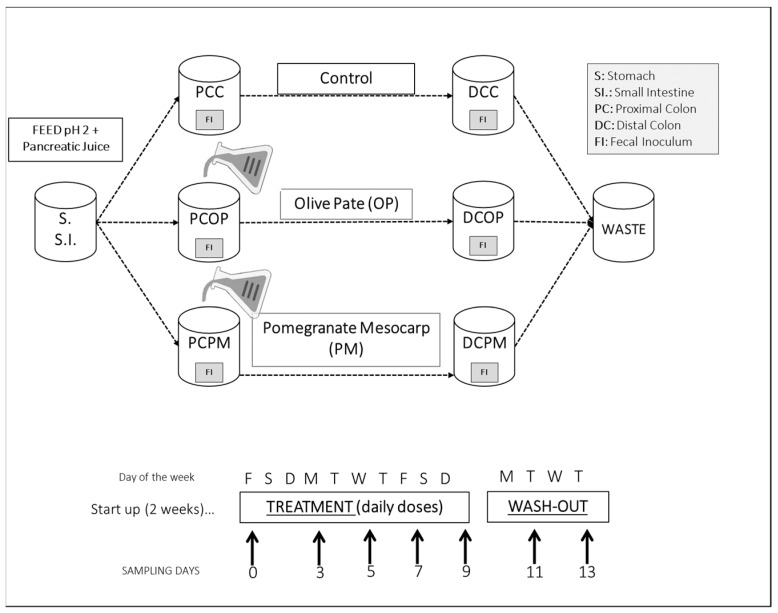
Applied experimental design with SHIME^®^.

In this study, the target was mainly to investigate the effect of the administration of repeated daily doses of the extract, simulating the usual intake of a food or dietary supplement for a long period. The phenolic composition of PM and OP is summarized in Table 1, where the fiber content of the two samples is also reported. The design of the experiment (Figure 1) requiring the use of two gastro simulators in a parallel way mimicked a long trial with one donor instead of multiple donors each with only one dose. The main phenols in OP were a group of oleuropein derivatives, free hydroxytyrosol, with minor amounts of verbascoside and luteolin, while the fiber content resulted in 20.4% of insoluble and 3.7% of soluble dietary fiber; monosaccharides and protein content were assessed at 16.8% and 9%, respectively. For PM, the analysis indicated α + β punicalagins as the main ellagitannins (70.7 mg/g), in co-presence with ellagic acid but in lower concentration (3.67 mg/g). Polysaccharide content in PM (soluble + insoluble fiber) was approximately 10%, and in accordance with the literature was mainly constituted by pectin [10,34,35,36].

### 2.3. Effects on Microbial Metabolism: SCFA and NH_4_^+^

**Figure 2:**
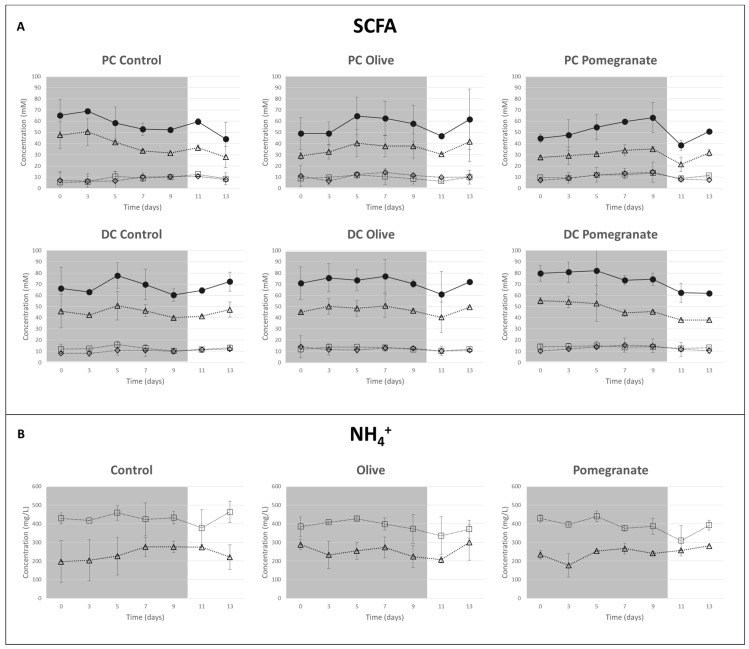
(**A**) Short chain fatty acid (SCFA) levels during and after treatment in PC and DC from control, OP- and PM-treated vessels in terms of acetate (∆), propionate (□), butyrate (◊), and total SCFA (●). (**B**) NH_4_^+^ levels during and after treatment in PC (□) and DC (∆), from control, OP-, and PM-treated vessels.

The metabolic activity of bacteria was investigated through quantitative analysis of SCFA and NH_4_^+^ concentrations, and the resulting charts are shown in Figure 2A as mean values.

SCFA levels showed quite a regular trend in control vessels, except for the intrinsic variability of the system. Overall concentrations in DC samples were higher than in PC ones, but the evolution was consistent; similar behavior occurred for NH_4_^+^ concentrations in both DC and PC vessels.

Samples from vessels treated with OP showed a trend comparable to the control, in both proximal and distal vessels, and no relevant changes were recorded during or after the treatment. As expected, according to the prevalent content of insoluble fiber, recognized as a non-fermentable or scarcely fermentable substrate, SCFA production was not modified with respect to the control. Only a small decrease was observed after the end of the treatment, particularly in DC, because interruption of administration affected functionality, which needed two days to go back to starting levels.

In PC vessels treated with PM, a weak increment in SCFA levels was observed up to nine days, with a physiological reduction after the end of administration and a new increase, close to starting values, after washout. In the DC vessel, only a weak but not relevant decrease of concentrations was highlighted. Differently from the OP extract, the PM decoction produced a weak prebiotic effect, probably due to its polysaccharides being mainly constituted by pectin that exhibited prebiotic properties in vitro [10,37]. Recent studies demonstrated the capacity of pectin to promote the growth of colonic *Bacterioidetes*, but only few data refer to *Firmicutes* [38,39,40]. Among the *Firmicutes*, the growth of *Eubacterium eligens* was promoted specifically by different apple pectin, which were hydrolyzed by a constitutive pectate lyase [40]. Studies on the specific effects of pectin from pomegranate fruits on human microbiota have not been available until now.

Over time, NH_4_^+^ levels were comparable to those of the control during treatment and washout for both OP and PM (Figure 2B), indicating the system was stable with only weak variability.

A preliminary experiment was also carried out using a no-carbohydrate medium in the SHIME^®^ vessels, and the SCFA production was studied (Supplementary Materials Figure S1). OP administered samples presented a different trend compared with the control: in both PC and DC, an increase of SCFA level was recorded during the treatment period and concentrations rose about 50%, meaning that OP was used as a carbon source by the bacterial community. Treatment with PM highlighted a similar trend; in the PC vessel SCFAs increased regularly up to nine days of feeding, and the levels of SCFAs rose in both PC and DC over the course of the experiment. This effect may again be due to polysaccharides in PM, used as a carbon source by the microbial population.

Overall, neither extract had any relevant effects on normal levels of SCFA and NH_4_^+^ after administration of a relatively high level of total phenols (390.4 mg/day for OP and 240.4 mg/day for PM). Compared to PC and DC in controls, only slight increases were recorded and no antimicrobial effect was observed at these doses. It can be affirmed that OP and PM do not affect normal microbial functionality.

### 2.4. Changes in Microbial Composition by Illumina

**Figure 3:**
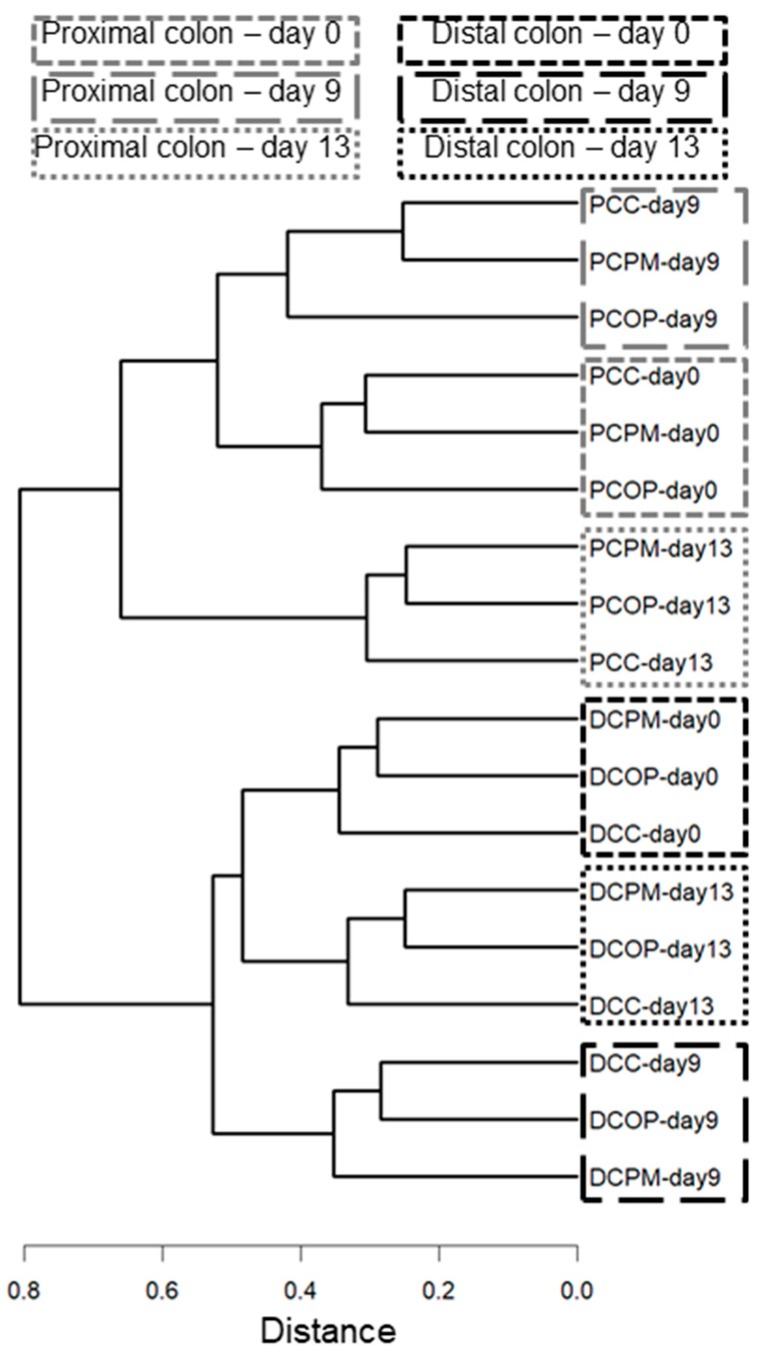
Cluster dendrogram of microbial composition from Illumina sequencing of SHIME^®^ samples (obtained as mean sample by the union of two replicas) after feeding with OP and PM extracts.

As shown in Figure 3, a clusterization was observed for the control and two samples at the same times both for proximal and distal samples, confirming that no significant changes occurred to microbial composition; the family distribution confirmed the information from cluster dendrograms (Supplementary Materials Figure S2).

**Figure 4:**
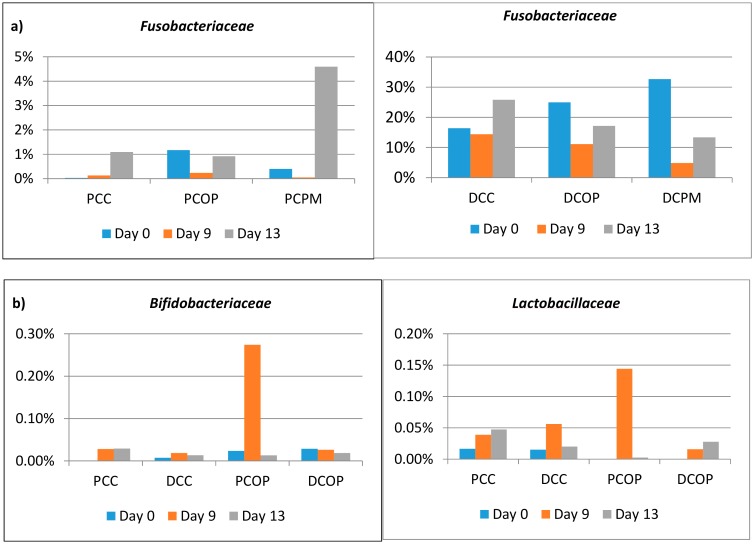

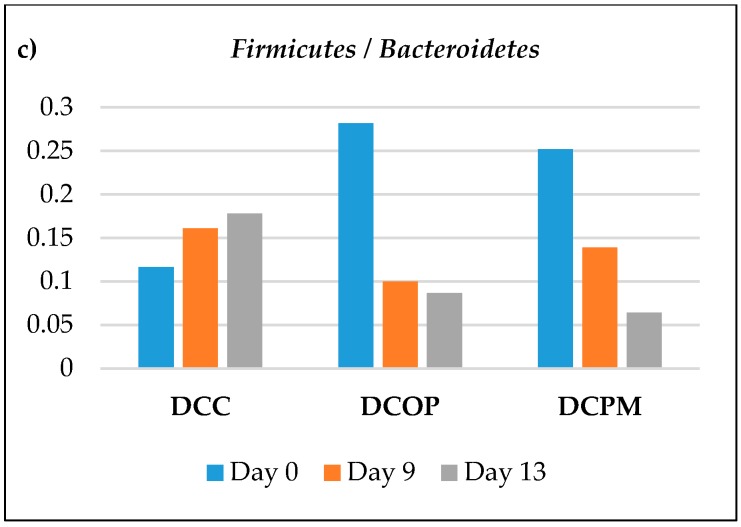
Families within lumen: (**a**) *Fusobacteriaceae* after feeding with OP and PM in proximal (PC) and distal (DC) tracts; (**b**) *Bifidobacteriaceae* and *Lactobacillaceae* after feeding with OP in proximal and distal tracts.; (**c**) Families within lumen, *Firmicutes*/*Bacteroidetes* ratio in distal tract after feeding with OP and PM.

Positive modifications were induced with subtle changes in relative abundance of certain bacteria, with a more relevant effect of PM with respect to OP, particularly after nine days of feeding. The relative abundance of different families in distal vessels treated with the two extracts indicated a reduction of *Fusobacteriaceae* (Figure 4a), a pro-inflammatory and invasive family associated with IBD and acute appendicitis [41].

A significant increase of *Lactobacillaceae* and *Bifidobacteriaceae* was observed only in PCOP (Figure 4b). Furthermore, the ratio *Firmicutes*/*Bacteroidetes* was influenced by both the extracts at the distal level, with a decrease of the value during treatment and washout (Figure 4c). The test carried out with the feeding mixture without sugars was applied to partially simulate a diet characterized by a low energy intake, often incorrectly proposed to reduce body weight. The evaluation of microbial functionality (Supplementary Materials Figure S1) showed lower concentrations in control samples compared with the complete feeding mixture (Figure 2), with an improvement in SCFAs provided by the administration of PM for nine days, not only in the PC vessel but also in the DC sample. This result could be attributable to the presence of the higher content of fermentable fiber (mainly pectin) in PM differently from OP, in which this is a minority fraction of the total dietary fiber (Table 1).

Increases in *Lactobacillaceae* and *Bifidobacteriaceae* (Figure 4b) suggested a certain specificity of the OP sample to enhance growth of these bacteria. Furthermore, the observed decrease of the *Firmicutes*/*Bacteroidetes* ratio in the distal tract (Figure 4c) highlights a potential benefit of these samples, since a high ratio between these families has been related to obesity risk [42].

The role played by the carbohydrate fraction in pomegranate decoction and olive pâté was preliminarily evaluated by testing no-carbohydrate feeding. The results, as shown in Supplementary Materials Figure S1, suggested that part of these polysaccharides was used as a carbon source by intestinal bacteria, in proximal and distal tracts, to maintain a stable level of SCFA and contrast the negative effects of extreme diets (high proteins/no sugars) on gut wellness.

### 2.5. Polyphenol Metabolic Fate

**Figure 5:**
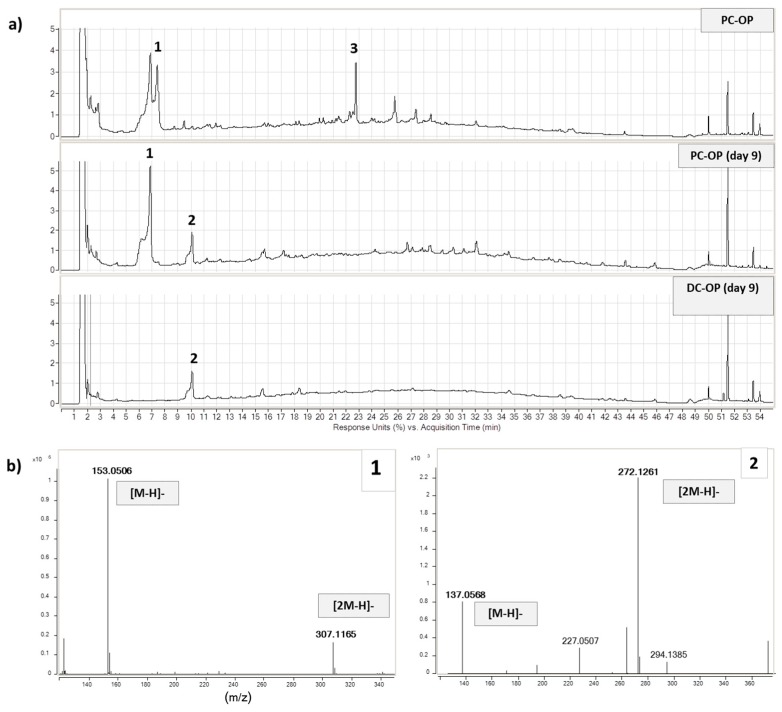
(**a**) Chromatographic profiles at 280 nm of vessel supplemented with OP extract at time 0 (PC-OP), and after 9 days of treatment (PC-OP day 9 and DC-OP day 9); hydroxytyrosol (**1**), tyrosol (**2**), and verbascoside (**3**). (**b**) MS spectra of compounds **1** and **2** with the dimeric forms.

Samples from different vessels and times (PC-OP day 9, DC-OP day 9, PC-PM day 9, and DC-PM day 9) and control samples (PC-OP and PC-PM) were treated before the analysis to precipitate soluble proteins and were analyzed by HPLC-DAD/MS-TOF with HP 1100 liquid chromatography coupled with HP 6200 series MS-TOF (Agilent Technologies, CA, USA). Hydroxytyrosol was detected in PC-OP (Figure 5a) according to its rt value, and mass spectrum (ions [M − H]^−^ at 153.05 *m*/*z* and [2M − H] ^−^ at 307.12 *m*/*z*). In the PC-OP day 9 sample, the concentration of hydroxytyrosol decreased and a new compound, corresponding to tyrosol (molecular ion at 137.06 *m*/*z*, dimer at 272.13 *m*/*z*), appeared in both PC and DC samples. Presumably due to its low concentration in OP (Table 1) and a possible fragmentation according to the scheme in Supplementary Materials Figure S3, verbascoside was undetectable, while by applying extract ion processing, the ion species at 179.15 *m*/*z* ascribable to caffeic acid was detected in PC-OP and DC-OP samples after nine days. Our evidence agrees with the presence of well-known esterases, which are normally active in microbiota and able to hydrolyze several phenolic compounds [43], such as verbascoside and secoiridoidic derivatives, both of ligstroside and oleuropein (producing free hydroxytyrosol and tyrosol). Analogously to verbascoside, the secoiridoidic derivatives were completely absent in all the vessels due to the esterase action, but also because they are very unstable molecules.

Hydroxytyrosol was partially metabolized at the proximal level, but was not detected in the distal sample, indicating a complete metabolization at this level. This result does not agree with Mosele and co-workers [31], who found hydroxytyrosol in fecal samples. Conversely, other groups [32] pointed out a significantly higher level of tyrosol in human plasma and urine after an intake of biscuits enriched with olive phenols. The same authors underlined how this metabolite partially derives from colonic metabolism. Our results from the SHIME^®^ system agree with these latter in vivo findings, confirming this system as a suitable tool to simulate the human colonic metabolism before intervention studies. Furthermore, the biotransformation of OP polyphenols by the gut microbial community can help to increase the antioxidant potential in the intestinal lumen due to the release of small phenols by esterase action. Hydroxytyrosol, which rapidly oxidizes as pure standard in water solution, was detected at the distal colon, confirming the stability of the molecule in anaerobic conditions.

**Figure 6:**
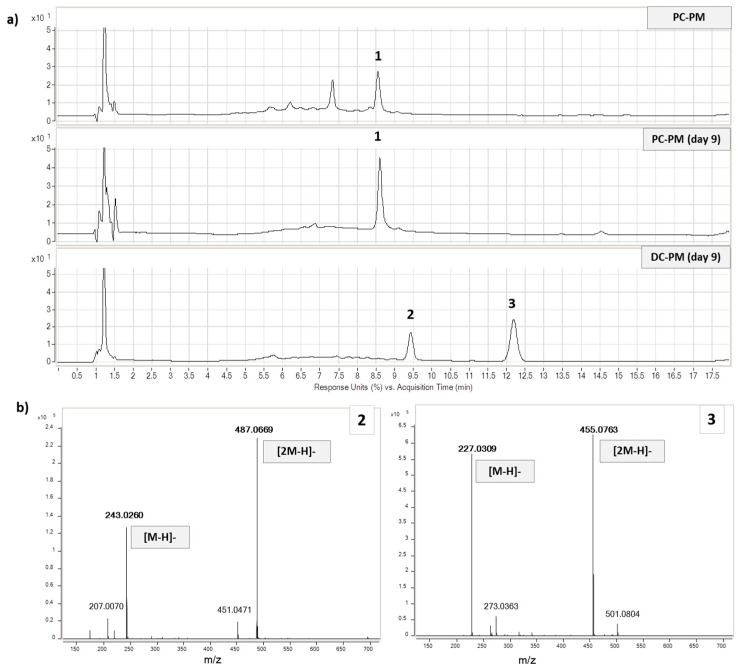
(**a**) Chromatograms of vessels supplemented with pomegranate, at time 0 (PC-PM), and after treatment (PC-PM day 9 at 370 nm and DC-PM day 9 at 280 nm). Ellagic acid (**1**), urolithin C (**2**), and urolithin A (**3**); (**b**) MS spectra of urolithin C (**2**) and urolithin A (**3**), both with the corresponding dimeric forms.

Regarding PM, the chromatographic profile of the proximal vessel after nine days of feeding (PC-PM day 9) showed again ellagic acid as the main phenol, as obtained at time 0 (PC-PM); ellagic acid was easily detectable at rt 15 min, by the [M − H]^−^ ion at 301 *m*/*z*. The absence of punicalagins, not detected in PC and DC vessels (Figure 6a) also at time 0, was correlated to their well-known ability to form insoluble complexes with soluble proteins [44] that are highly included in liquid luminal suspension. In any case, punicalagins (free or complexed with proteins) interacted with the colonic microbiota because in DC two main metabolites of these ellagitannins were identified: urolithins C and A (Figure 6a).

As expected, the profile of the distal colon, derived after a longer exposure to microbiota metabolism (DC-PM day 9), was different. The ellagic acid disappeared, and two new metabolites were detected and identified (Figure 6A and Supplementary Materials Figure S4), according to UV-Vis spectra and literature data [16], as urolithin A and urolithin C. Their mass spectra (Figure 6B), showing the molecular ions (at 227.03 *m*/*z* and 243.03 *m*/*z* respectively) and the corresponding two dimers ([2M − H]^−^), further confirmed the identification. Our result is consistent with previous in vivo studies that showed the metabolic path of pomegranate ellagitannins to urolithins in the distal intestinal tract [28,45]. Once again, the SHIME^®^ system was effective in identifying the metabotype of the human donor [16]. Only urolithins C and A were produced in the distal tract, without traces of urolithin B (Figure 6), indicating the presence of metabotype A. Compared to Garcia-Villalba et al. [16], in our work the composition of the tested sample (PM) was very different. In particular, a lower dose of punicalagin was administered per day (30% less) and for a shorter time (nine instead of twenty one days), the amount of ellagic acid and its glycosides was nine times lower (20 mg instead of 180 mg per day), and finally approximately 200 mg of polysaccharides per day were present in the sample and were administered. In agreement with García-Villalba et al. [16], the punicalagins, although in higher amounts and administered for a longer time, were not detected in the distal tract. At the same time, unlike García-Villalba et al. [16], we observed little increase in SCFAs in the proximal tract (Figure 2A), presumably due to the co-presence of pectin, but also to a lower amount of ellagitannins (both as quantity/day and total days of administration), which could exert moderate antimicrobial effects at high doses. Furthermore, we observed how a carbohydrate-free diet associated with a lower dose of ellagitannins was again able to show an increase in SCFA production over time (Supplementary Materials Figure S1).

## 3. Materials and Methods

### 3.1. Pomegranate and Olive By-Products

Pomegranate fruits (Wonderful cv) cultivated on farms in Tuscany, were harvested in Grosseto (Tuscany, Italy). The mesocarp was manually separated from the other parts, then a 1 h decoction was applied [10]; the suspension was freeze-dried, ground, and used as powder (PM). The olive “pâté” was obtained from a milling process with biphasic decanter and a final separator “Leopard” (Pieralisi, Italy); the sample, including only wet pulp and husk, was freeze-dried. The powder was washed by adding n-hexane (15 mL/g) and stirring for 1 h to remove lipid fractions, and the residual powder was used for the experiments (OP).

Polysaccharide content was determined after ethanol precipitation, and in accordance with the literature was mainly constituted by pectin [10,34,35].

### 3.2. Standards

The following pure standards were used for qualitative and quantitative analyses: α + β punicalagins, ellagic acid, hydroxytyrosol, tyrosol, oleuropein, and caffeic acid, all purchased from Sigma Aldrich (St. Louis, MO, USA).

### 3.3. SHIME^®^ Experiments

Two replicates of a triple SHIME^®^ experiment were performed at different times, using fecal samples from the same healthy donor as inoculum to simulate a traditional luminal microbial community [46]. The SHIME^®^ also contained mucin microcosms (K1-carrier, AnoxKaldnes AB, Lund, Sweden), submerged in mucin-agar to host surface-attached microbes [47]. The feed selected included (g/L): arabinogalactan (1), pectin (2), xylan (1), D-(+)-glucose (0.4), starch (4), yeast extract (3.0), peptone (1.0), and pig gastric mucin (4.0). Proximal colon (PC) vessels were filled with 500 mL of feed and 80 microcosms, while distal colon (DC) units were filled with 800 mL of feed and the same amount of mucin. Inoculation was performed with 40 mL of a 1:5 dilution of fresh stools [48]. Three series of colon vessels (proximal and distal) ran simultaneously: one pair for control, one for OP, and the third for PM treatment (Figure 1). After 18 h incubation for pH stabilization, 140 mL of nutritional medium and 60 mL of pancreatic juice were supplied to each colon compartment three times per day. The system was kept at 37 °C under anaerobic conditions. In the first 2 weeks the system was stabilized, then daily doses of the extract were administered to the PC vessel for 10 days, while the control PC did not receive any administrations in the same period. At the end of the treatment, a 4 day washout was carried out. Experiments were performed with different extract dosages: 4 g/L of OP and 2 g/L of PM. Three times per week, at the same time every day, 20 mL of liquid sample were collected from each colon vessel (Figure 1). Then 1 mL was centrifuged, and the pellet saved. All samples were stored with residual liquid at −20 °C.

#### Tests with No-Carb Diet

The experiments described above, using the same amount of the two extracts (4 g/L of OP and 2 g/L of PM), were also carried out providing a feed without a carbohydrate fraction. The SHIME^®^ contained a special feed without sugars included: yeast extract 3.0 g/L, peptone 1.0 g/L, and pig gastric mucin 4.0 g/L.

For each experiment, after a stabilization period of 2 weeks, daily doses of extracts were administrated directly to the PC vessel for 10 days.

### 3.4. HPLC/DAD/MS Analysis of Phenolic Compounds

Solvents and standards of analytical purity were purchased from Sigma Aldrich (St. Louis, MO, USA). The control samples (PCC) were added with fresh extracts at the same concentrations used for experiments, to evaluate starting point profiles (PCC_OP and PCC_PM). These samples were then compared with proximal and distal samples recovered during treatment with extracts (PCOP_d9, DCOP_d9, PCPM_d9, and DCDM_d9). Samples of vessel content collected before the administration were also analyzed to evaluate possible interferences due to feed components.

All samples were pre-treated in order to obtain a clear solution, adding a mix of acetone/acetonitrile/methanol 1:1:1, as described previously [49]. Samples from vessels administered with PM received the same treatment, but the original supernatant was also acidified with HCOOH. The suspension was then centrifuged at 4 °C, 5000 rpm, 10 min. Then, 1 mL of supernatant was recovered and dried using N2 gas. The dried residue was dissolved in 200 µL of ethanol/acidic water (by HCOOH) 7:3 *v*/*v*. Final ultracentrifugation (10 min, 14,000 rpm) was applied to obtain clear samples for HPLC. The analyses were performed using an HP 1100 liquid chromatography (Agilent Technologies, USA) coupled with HP 6200 series MS-TOF.

For OP treated samples, a 150 mm × 3 mm i.d., 2.7 μm, RP-18, Poroshell column (Agilent Technologies, USA) was used. Eluents selected were (A) H_2_O at pH 3.2 by formic acid and (B) CH_3_CN. The multi-step linear solvent gradient used was: 0–40 min 5–40% B; 40–45 min, 40% B; 45–50 min 40–100% B; 50–53 min 100%; 53–55 min 100–5%, equilibration time 10 min; flow rate 0.4 mL/min. The UV–Vis spectra were recorded in the range 200–600 nm and the chromatograms were acquired at 240 nm, 280 nm, 330 nm, and 350 nm. MS spectra were acquired using Dual-ESI source in negative polarity, with an 80 V fragmentor, 3800 V capillary voltage, 350 °C of gas temperature. For quantitative analysis of OP samples, hydroxytyrosol and tyrosol were quantified using a calibration curve of tyrosol standard with R² = 0.999 at 280 nm. Oleuropein derivatives (280 nm) were quantified by applying a four-point calibration curve obtained with standard oleuropein with R² = 0.999. Verbascoside and derivatives were determined using a caffeic acid calibration curve at 330 nm with R² = 0.999.

PM treated samples were analyzed using a 150 mm × 2 mm i.d., 4 μm, RP-18, Sinergi Fusion column (Phenomenex, Torrance, CA, USA). As seen for OP samples analysis, (A) H_2_O at pH 3.2 by formic acid and (B) CH_3_CN were selected as eluents. The multi-step linear solvent gradient used was: 0–4 min 5–25% B; 4–8 min, 25–25% B; 8–14 min 25–35% B; 14–16 min 35–90%; 16–18 min 90–5%, equilibration time 10 min; flow rate 0.4 mL/min. The UV–Vis spectra were recorded in the range 200–500 nm and the chromatograms were acquired at 240 nm, 280 nm, 330 nm, 370 nm, and 380 nm. MS spectra were acquired using Dual-ESI source in negative polarity with a 100 V fragmentor, 4000 V capillary voltage, 350 °C of gas temperature. Quantitative analysis of PM components was performed using α + β punicalagins (380 nm) in a linearity range between 0.5 and 8 μg with an R² = 0.9994; the calibration curve of ellagic acid (370 nm) was in the linearity range of 0.031–1.25 μg with an R² = 0.9995.

### 3.5. SCFA and NH_4_^+^

For SCFA analysis, a liquid-liquid extraction with diethyl ether was used, after the addition of H_2_SO_4_ and internal standard. SCFA quantitative analysis was performed by capillary gas chromatography coupled with a flame ionization detector (GC-FID), as previously described [50].

Ammonium levels were analyzed by steam distillation according to standard methods 4500-NH3 B [50]. Determination of total ammoniacal nitrogen (TAN) in liquid luminal samples was performed through NH_4_^+^ quantification by the addition of MgO, distillation of NH_3_ into boric acid solution and subsequent back-titration.

SCFA and NH_4_^+^ values obtained from the two experiments were reported as average ± standard deviation.

### 3.6. DNA Extraction

Bacterial DNA from luminal samples was extracted as described earlier [51], using a lysis buffer (TrisEDTA, NaCl, PVP40, SDS, water, (Milford, MA, USA)) and glass beads for FastPrep. Extraction was performed with phenol-chloroform, and EtOH/NaOAc was used for precipitation [52]. Samples were dissolved in TrisEDTA 1X and stored at −20 °C. Concentration and quality were verified by Nanodrop (Thermo Scientific, Beverly, MA, USA) and 2% agarose gel electrophoresis.

### 3.7. Illumina Sequencing Samples from SHIME

The samples from the two experiments were pooled and the V5–V6 hypervariable regions of the 16S rRNA gene were PCR-amplified and sequenced by MiSeq Illumina (Illumina, Inc., San Diego, CA, USA) using a 250 bp × 2 paired-end protocol. The multiplexed libraries were prepared using the 783F and 1046R primers [53,54]. The PCR was performed in 2 × 50 µL reactions with GoTaq Green Master Mix (Promega Corporation, Madison, WI, USA) and 1 µM of each primer as previously reported [55]. The amplicons were purified with the Wizard SV Gel and PCR Clean-up System (Promega Corporation, Madison, WI, USA,) according to the manufacturer’s instructions, and quantified using Qubit (Life Technologies, Carlsbad, CA, USA). All DNA samples were tested for amplification inhibition by sample dilution. DNA sequencing was carried out at Parco Tecnologico Padano (Lodi, Italy). Reads from sequencing were demultiplexed and bioinformatic elaborations were performed as previously reported [56]. Operational Taxonomic Units (OTUs) were defined on the whole data set clustering the sequences at 97% sequence identity and defining a representative sequence for each cluster. The abundance of each OTU was estimated by mapping the sequences of each sample against the representative sequence of each OTU at 97% sequence identity. Classification of the sequences representative of each OTU at different taxonomic ranks was done using the RDP classifier (≥80% confidence) [57]. A cluster analysis (CA) based on Hellinger transformed family relative abundance data was performed using the vegan package [58] in R 3.5.1 [59].

## 4. Conclusions

These by-products from olive and pomegranate food processing, characterized by the co-presence of phenolic components and polysaccharides, were evaluated for the first time for their interaction with human microbiota after repeated administration using the in vitro SHIME^®^ system. We demonstrated that the selected doses of olive and pomegranate, after repeated daily administration, do not have antimicrobial effects on the colonic microbial community because the SCFAs production was not reduced. On the contrary, they determined some positive changes, such as the reduction of *Fusobacteriaceae* (associated with an inflammatory status) or the net increase in *Lactobacillaceae* and *Bifidobacteriaceae*, as in the case of olive pâté. Even in a more critical condition, such as a no-carbohydrate diet, microbial functionality and gut wellness were maintained as confirmed by the SCFA production. Regarding the pomegranate extract, the structure of the urolithins produced in the SHIME^®^ system confirmed the presence of metabotype A. Furthermore, the fate of polyphenols included in olive pâté was found to be congruous with enzymatic pathways already observed in in vivo studies; tyrosol as main metabolite in distal colon was highlighted for the first time. Although this in vitro system clearly has limitations in reproducing all the interactions and mechanisms involved in the human gut, the obtained information is consistent with results from previous in vivo studies.

Our data suggested that the repeated administration of these samples, obtained from agricultural by-products using simple and green processes, can be suitable as new functional food ingredients to provide a daily intake of beneficial polyphenols and fermentable polysaccharides as in the case of pomegranate extract. Further in vivo studies on standardized samples obtained as blends of extracts from more batches of olive pâté and pomegranate peel are required to demonstrate if the tested by-products are safe for administration in dietary supplements.

## Figures and Tables

**Table:** (**a**)

Sample	Phenols	mg/g DW
OP	Verbascoside	4.52
	Hydroxytyrosol	15.3
	Oleuropein derivatives	77.8
	Luteolin	0.42
	Total polyphenols	97.6
PM	α + β punicalagins	70.7
	Ellagic acid and derivatives	10.7
	Total ellagitannins	120.2

**Table:** (**b**)

	Applied Method	OP	PM
Insoluble fiber	AOAC Official Method 991.43 (Total, Soluble, and Insoluble Dietary Fiber in Foods)	20.4%	3.0%
Soluble fiber		3.7%	6.7%
Proteins	ISTISAN 96/34 Analytical methods used in food chemical control	9%	2.3%
Total sugars		16.8%	44%

## Supplementary Materials

The following are available online: Figure S1. SCFA levels; Figure S2. Microbial distribution, and Figure S3, S4: Chemical structure of the main phenols.
