# Supplementary material for: Effects of Olive and Pomegranate By-Products on Human Microbiota: A Study Using the SHIME® In Vitro Simulator
Source: Molecules. 2019 Oct 21;24(20):3791. doi: 10.3390/molecules24203791 (PMC6832639; doi:10.3390/molecules24203791)
Supplement: Supplementary file 1 [file molecules-24-03791-s001.zip › molecules-602291-SI.pptx]

## Slide 1
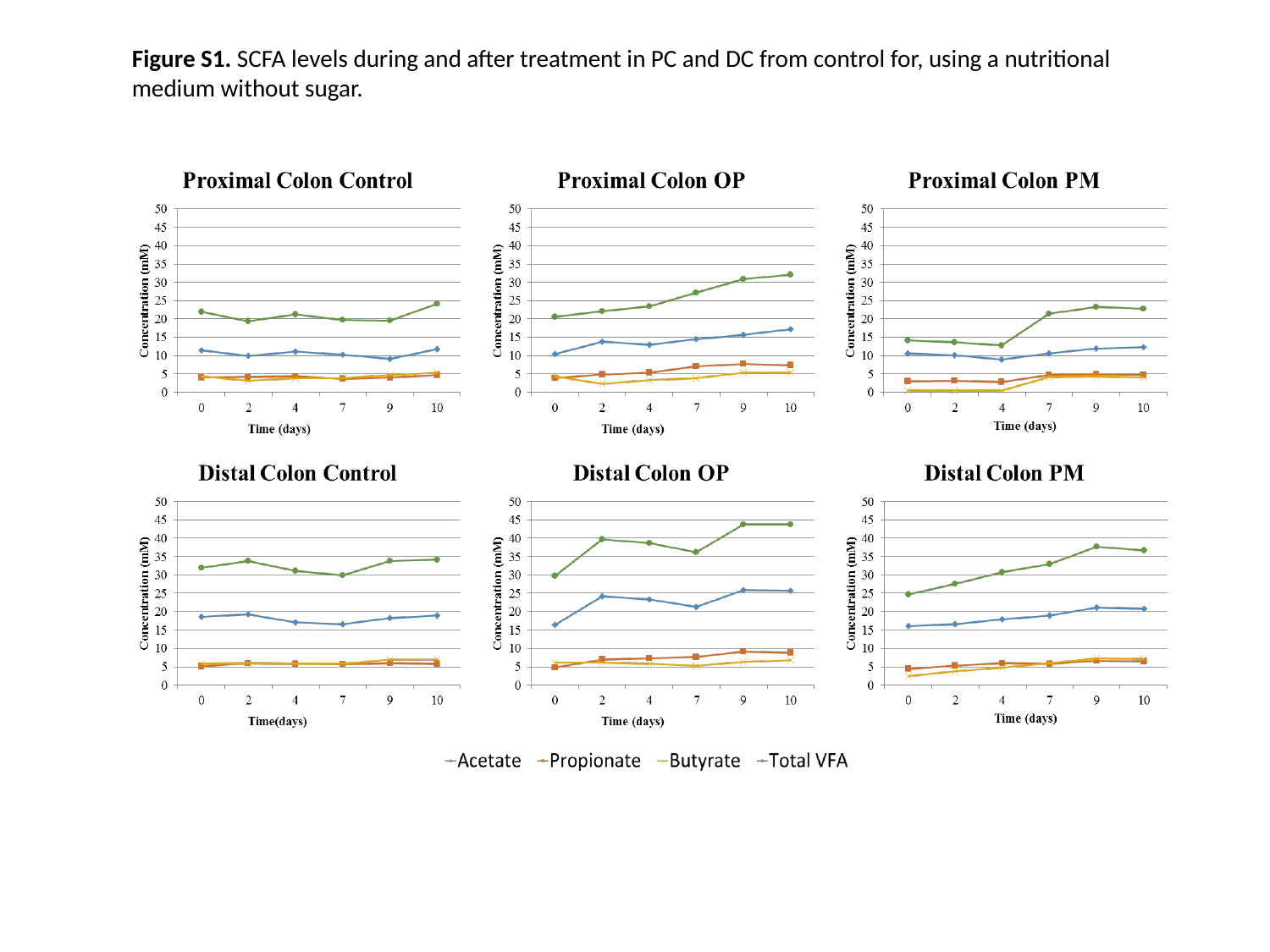

Figure S1. SCFA levels during and after treatment in PC and DC from control for, using a nutritional medium without sugar.

## Slide 2
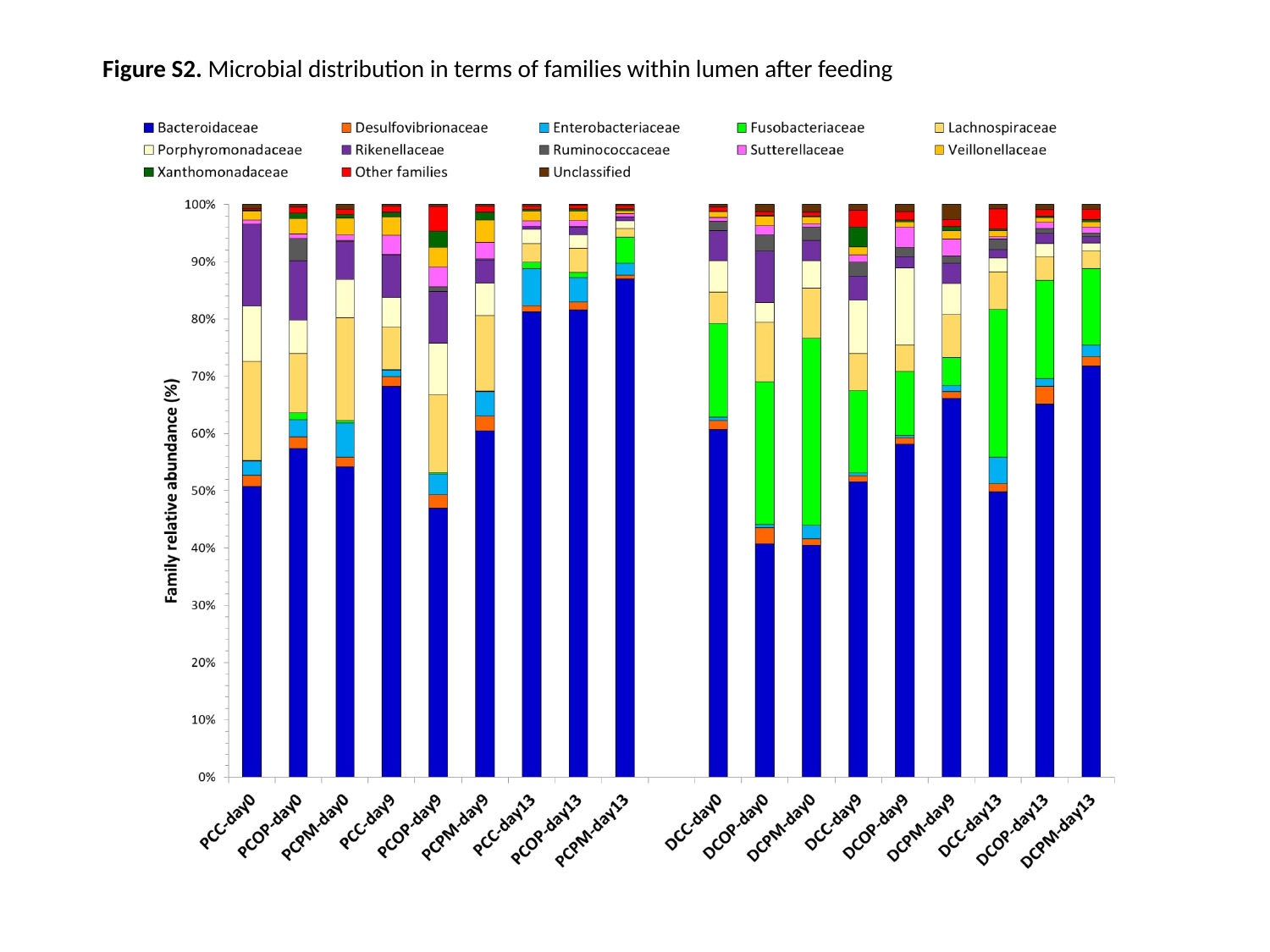

Figure S2. Microbial distribution in terms of families within lumen after feeding

## Slide 3
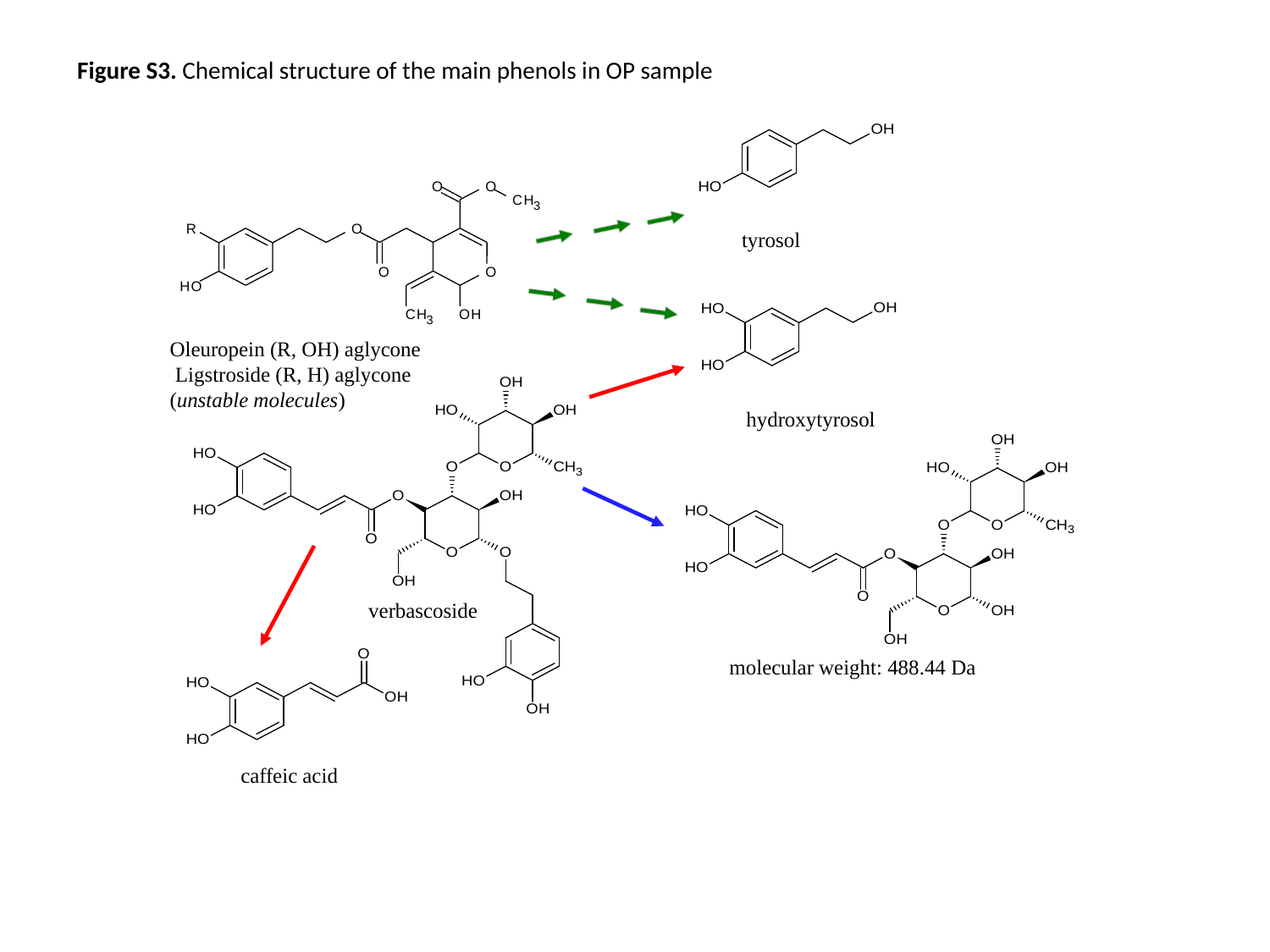

Figure S3. Chemical structure of the main phenols in OP sample
O
O
C
H
3
O
R
O
O
H
O
C
H
O
H
3
tyrosol
Oleuropein (R, OH) aglycone
 Ligstroside (R, H) aglycone
(unstable molecules)
hydroxytyrosol
verbascoside
molecular weight: 488.44 Da
caffeic acid

## Slide 4
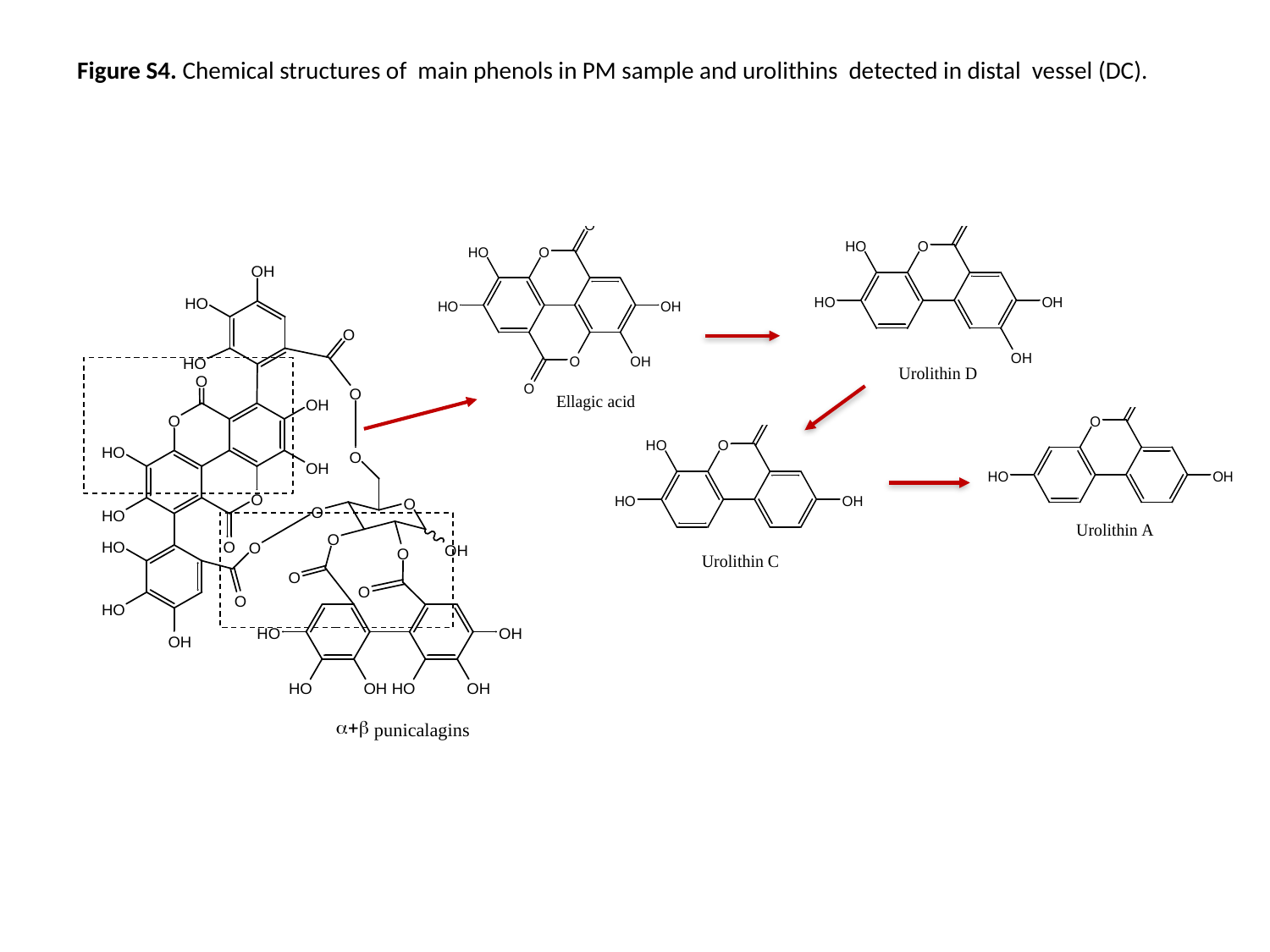

Figure S4. Chemical structures of main phenols in PM sample and urolithins detected in distal vessel (DC).
